# Factors Obscuring the Role of *E. coli* from Domestic Animals in the Global Antimicrobial Resistance Crisis: An Evidence-Based Review

**DOI:** 10.3390/ijerph17093061

**Published:** 2020-04-28

**Authors:** Fernanda Loayza, Jay P. Graham, Gabriel Trueba

**Affiliations:** 1 Microbiology Institute, Colegio de Ciencias Biológicas y Ambientales, Universidad San Francisco de Quito, Diego de Robles y Pampite, Cumbayá-Quito P.O. BOX 170901, Ecuador; 2 Berkeley School of Public Health, University of California, 2121 Berkeley Way, Room 5302, Berkeley, CA 94720-7360, USA

**Keywords:** commensal *E. coli*, antimicrobial resistance, food-animals, gene transfer

## Abstract

Recent studies have found limited associations between antimicrobial resistance (AMR) in domestic animals (and animal products), and AMR in human clinical settings. These studies have primarily used *Escherichia coli*, a critically important bacterial species associated with significant human morbidity and mortality. *E. coli* is found in domestic animals and the environment, and it can be easily transmitted between these compartments. Additionally, the World Health Organization has highlighted *E. coli* as a “highly relevant and representative indicator of the magnitude and the leading edge of the global antimicrobial resistance (AMR) problem”. In this paper, we discuss the weaknesses of current research that aims to link *E. coli* from domestic animals to the current AMR crisis in humans. Fundamental gaps remain in our understanding the complexities of *E. coli* population genetics and the magnitude of phenomena such as horizontal gene transfer (HGT) or DNA rearrangements (transposition and recombination). The dynamic and intricate interplay between bacterial clones, plasmids, transposons, and genes likely blur the evidence of AMR transmission from *E. coli* in domestic animals to human microbiota and vice versa. We describe key factors that are frequently neglected when carrying out studies of AMR sources and transmission dynamics.

## 1. Introduction

The rapid evolution of antimicrobial resistance (AMR) in bacteria is one of the most dangerous trends in public health [1,2] causing increased morbidity, mortality [1,3,4,5,6,7,8], and economic loss [9]. The AMR crisis is being felt more intensely in hospitals where outbreaks of pan-resistant opportunistic pathogens are emerging at an increasing pace [10,11,12,13]. Most of these drug-resistant opportunistic pathogens found in clinical settings are members of the human (or other animal) commensal microbiota [14,15,16]. AMR in bacteria from food-animals has been reported since the 1950s when antimicrobial supplements began to be used as growth promoters in animal feed [17,18,19,20,21]. Currently, 75% of antimicrobials produced in the world are used in food-animals [22]; both small-scale food-animal producers and intensive food-animal operations use a variety of antimicrobials in animal feed and water as growth promotors or prophylactics [23,24,25,26,27]. Food-animal performance is an important issue in the food-animal industry and antimicrobials are perceived as a means to prevent disease or improve weight gain and feed conversion efficiency [28,29]. Antimicrobial use in this setting, however, causes selective pressure on the bacterial populations which accumulate AMR genes [30,31,32,33,34,35], and the large numbers and diverse AMR genes in the microbiota of domestic animals has created concern about the spread of AMR from food-animals to humans [13,36,37,38].

Transmission of AMR bacteria can occur through the environment [39] and food-chain [40,41,42], especially in low- and middle-income countries (LMICs) where water, sanitation, and hygiene are inadequate [43,44,45]. We focus on the role of commensal *E. coli* in the AMR crisis for three reasons. First, *E. coli* is probably the most studied indicator [46,47] and its transmission can be tracked more easily (among animal hosts) than anaerobes, which are the most abundant members of the microbiota [48,49]. Second, *E. coli* can survive and even grow in the environment outside of the host [50]. Third, *E. coli* may mobilize AMR genes more easily than other intestinal bacteria (such as *Bacteroides*) [51,52,53].

In this review, we focus on recent reports showing a lack of relationship between AMR in domestic animals and antimicrobial resistant bacteria in humans. We postulate that the complexities, due to high diversity, strain turnover, and horizontal gene transfer, hamper our ability to find greater linkage between AMR in domestic-animals and humans. We include pets in this study to show how an antimicrobial resistant *E. coli* can colonize different hosts. We analyze all the potential pitfalls associated to these types of studies. To minimize the potential overestimation of human–domestic animal transmission, we focus on reports in which whole-genome sequencing (WGS) was used, as multi locus sequence typing (MLST) can show homoplasious sequence-types [54] or strains belonging to the same sequence-type may show many single nucleotide polymorphisms (SNPs) in other genes indicating non-recent ancestry [55].

### Population Genetics of E. coli and AMR

*Escherichia coli* is found almost exclusively in the intestines of warm-blooded animals and although it represents only around 1% of the intestinal microbiota [56], it is probably the most abundant member of the intestinal microbiota possessing the ability to survive and even grow outside the host [50]. Commensal *E. coli* is probably the most common commensal bacteria transmitted among different species of animals [48,49]. Each *E. coli* strain falls into one of the six phylogenetic groups (A, B1, B2, D, E, or F) [57]. The majority of *E. coli* clones can colonize the intestines of different animal species (generalists), however, different *E. coli* strains may display a different degree of host adaptation, and the strains belonging to some phylogroups may be better adapted to certain animal species [57,58,59,60]. *E. coli* strains with a higher degree of adaptation to a given intestinal milieu may become long-time colonizers (residents) [61] and numerically dominant [62], while strains with lower adaptation may colonize transiently and/or may become a numerical minority. Numerically dominant and resident lineages may disseminate more between different hosts. The constant competition between new arrivals with colonizing strains in the intestine is likely responsible for the rapid turnover of dominant *E. coli* strains observed in the intestines of humans [63]. Although a minority among *E. coli* lineages, pathogenic strains of *E. coli* (such as ST131 genotype) are an important category that contains virulence genes and are associated with invasive infections. Antimicrobial resistance is another layer of complexity; the transmission of AMR genes among strains of *E. coli* occurs through the movement of mobile genetic elements (MGEs; e.g., plasmids, phages, transposons, integron-cassettes, and other mosaic structures) [33,64,65]. Transposable elements and cassettes (integrons) mediate the movement of AMR genes from one MGE to another or from a bacterial chromosome to plasmids (or vice versa), whereas plasmids mediate the movement of AMR genes from one bacterium to another [33,66,67]. This phenomenon is very dynamic; it is possible to find isolates that are the same *E. coli* clone, in the same intestine with different AMR genes [68,69]. All these categories (dominant, pathogenic, and antimicrobial resistant) are very fluid as *E. coli* strains may change their status by acquiring genes (horizontal gene transfer-HGT and recombination) or by mutations.

## 2. Materials and Methods

### 2.1. Study Population and Outcome of Interest

For this review, we considered relevant peer-reviewed literature that studied humans, farm animals, and pets carrying antimicrobial resistant commensal *E. coli*.

### 2.2. Identifying the Relevant Literature

The peer-reviewed literature was searched using Google, Google Scholar, MEDLINE, and PubMed using the keywords: farm animals OR domestic animals AND antimicrobial resistance OR antibiotic resistance AND *Escherichia coli* OR *E. coli* AND human.

### 2.3. Eligibility Assessment

Selected articles were submitted to an initial screening to determine the relevance based on title, abstract, and keywords. A second full-text screening was performed to analyze methods. Those that reported whole-genome sequencing for comparison of interspecies transfer of *E. coli* or AMR determinants were selected (Table 1).

## 3. Results and Discussion

### 3.1. Why Have Studies Failed to Show a Link between Antimicrobial Resistance in Humans and Domestic Animals?

Different sampling protocols often yield different results, and below we describe critical aspects of studies that can affect study findings. We note here that none of the studies included in this analysis reported to receive funding from sources that may have financial interests at stake, and all of the authors declared no conflict interest (Table 1).

### 3.2. Inadequate Sampling

Many studies have failed to find a clonal relationship or AMR gene homology between AMR *E. coli* obtained from humans in hospitals (opportunistic pathogens) and domestic animals [39,70,71,72,73]. When commensal isolates were obtained from domestic animals and humans living in proximity and during the same period, however, isolates were identified that showed clear clonal relationships and the same AMR genes in *E. coli* from humans and domestic animals [74,75,76,77]. We argue that reports analyzing isolates from different locations or different time frames underestimate the *E. coli* diversity and population dynamics. Populations of *E. coli* collected from different locations and different time frames are most likely different. Despite this fact, some reports have been able to find clonal relationships between infections in hospitalized humans and fecal samples from domestic animals [74,78]. We found one exception where commensal *E. coli* from domestic animals did not show clonal similarity to human *E. coli* in the same community and during the same time period [79]. An alternative interpretation of the discrepancies between studies is that *E. coli* from domestic animals transmit to humans through the environment (people working on farms or who are in contact with animals or their waste) and not through the food-chain [39], however, it seems more likely for an enteric bacteria (like all zoonotic enteric pathogens) to enter the human gut through food than any other route.

### 3.3. Focus on Opportunistic Pathogens

The bulk of the *E. coli* transmitted from domestic animals to humans are probably numerically dominant commensals, not frank pathogens. Numerically dominant *E. coli* commensals are lineages representing the majority [62]. Pathogenic strains of *E. coli* make up a limited number of the *E. coli* lineages which may be moving from domestic animals to humans; these pathogens, however, are probably a minority in many animal intestines. Therefore, it is no surprise that some studies failed to detect some opportunistic human pathogens (such as *E. coli* ST131) in fecal samples from domestic animals or animal products [39,70,71]. If these strains are part of the commensal *E. coli* in domestic animals, they are experiencing the same population fluctuations associated with clonal competition (described above). Assessing the prevalence of pathogenic *E. coli* in domestic animals or animal products may require massive sampling and metagenomic approaches. Nevertheless, some studies have been able to detect the same clones of opportunistic pathogens in hospitals and domestic animals or food-animal products [42,74,75].

### 3.4. Complex Dynamics of Mobile Genetic Elements

**Figure 1:**
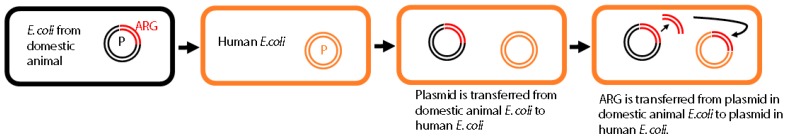
Example of antimicrobial resistance gene (ARG) movement that affects the complexity of studying antimicrobial resistance transmission. Plasmids (P) carried by *E. coli* from a food-animal can be transferred to human *E. coli* and the ARG can move between plasmids.

Transmission of AMR genes between domestic animal microbiota and human microbiota seems to occur more frequently by HGT than clonal transmission [39,53,68,77,78,80,81,85]. Nevertheless, the HGT of AMR genes complicates the identification of the source of AMR genes. It is possible that AMR *E. coli* strains (e.g., from a domestic animal) marginally colonize the human gut, but it may transfer a plasmid to a dominant bacterial strain (human-adapted) in the human intestine [68,78,86] and the same AMR gene may move via a transposon (or cassette) from the mobilized plasmid (from an animal bacteria) to a plasmid in the human bacterium [33,66] (Figure 1). Under this scenario, only a longitudinal analysis including whole plasmid sequencing of epidemiologically related (spatiotemporally linked) strains could capture this phenomenon. Identical AMR genes are carried by different *E. coli* plasmids in diverse isolates obtained from humans and domestic animals living in the same community and during the same period [55]. Recent studies (using WGS and plasmid sequencing of epidemiologically related isolates) show how transposable elements restructure plasmids with AMR genes in bacterial strains that are causing infections in one hospital over time [66], and how some plasmids can undergo rearrangements in a short period of time [67]. Plasmids carrying AMR genes also have a different ability to disseminate, such that many exhibit different levels of bacterial host specificities and cause different fitness costs in different bacterial populations [87]. Due to the complexity of the phenomena involved, the transmission of AMR genes from food-animal *E. coli* to human *E. coli* may not be possible to demonstrate molecularly but only epidemiologically (i.e., *E. coli* strains isolated from epidemiologically related sources, have the same AMR genes) [55,82,83,88,89].

### 3.5. Focus on Non-Dominant Clones

Results from studies carried out on strains isolated with non-selective media (e.g., containing no antimicrobials) will differ from studies in which *E. coli* was isolated in media with antimicrobials. In the first case, we are likely assessing the numerically dominant *E. coli* [62], while in the second case we may be looking at a minority *E. coli* lineage with a specific resistance phenotype. As previously mentioned, numerical dominance may be related to some degree of adaptation of some *E. coli* lineages to an animal host; some generalist strains can thrive similarly in the intestines of different animal species, while others likely thrive in one animal species rather than in others [58,60]. We argue that a specialist *E. coli* strain colonizing the intestine of a host, for which it is not adapted, may remain a numerical minority [58] and undetectable by standard bacteriological culturing techniques (e.g., collecting 5–20 colonies from a culture plate) [62]. Conversely, when a strain is more adapted to the host, it is likely to become a numerically dominant lineage and easily detected by standard bacteriological culture. This property may then indicate that human to human *E. coli* transmission is more frequent than the transmission of *E. coli* from domestic animals to humans because of a higher exposure of human populations to human strains [72].

### 3.6. Different Environmental Contexts

Other factors responsible for discrepancies between studies may be associated with the environmental setting; industrialized countries have better environmental and food hygiene and sanitation than low- and middle-income countries (LMICs), and some differences in *E. coli* transmission should be expected in different contexts. Similar considerations must be made when comparing rural (farming communities) vs. urban communities [72,74,77]. For food-animal operations in low-income countries, or where there is insufficient biosecurity and hygiene in the facilities, reducing the use of antimicrobials is perceived as a big challenge [90,91].

The review of the literature indicates that there is little doubt that cross-colonization of AMR bacteria from domestic animals to humans is occurring and many studies have shown this. There is also compelling evidence that AMR genes that originated in food-animals can end up in *E. coli* strains that reside in the human gut [39,55,77,83]. However, in some instances, these phenomena may not be evident because the large diversity and constant turnover of *E. coli* strains in the intestines reduces the chances of finding a link, especially when sampling from different locations or during different timeframes as was done in many previous studies. The movement of AMR genes from one plasmid to another, or plasmids undergoing rearrangement, are also important obstacles to understanding the linkage between AMR in human and domestic animals [67].

Horizontal gene transfer acts as a mechanism that can quickly spread resistant determinants to new carriers regardless of whether they are human or animal linages of bacteria [83,92]. AMR genes and the MGEs that mobilize these genes are likely to be derived from diverse parts of the microbial biosphere [13,93]. The gut microbiome has been defined as an important source of AMR genes in both animals and humans [94], and the dynamic nature of the gut is likely complicated further by the dynamics of HGT [95,96].

There are likely major differences in the transmission of AMR in high-income, middle-income, and low-income countries. For example, poor hygienic conditions in the food-animal industry in low-income countries may accelerate the transmission of bacteria from food-animals through the food-chain; lack of wastewater treatment or lack of basic sanitation infrastructure may contaminate irrigation water or soil where crops are raised [97,98]. The latter transmission pathway is also troublesome as AMR may return to humans via food-animals and the food-chain. One potential example of this phenomenon is carbapenem resistance, which is thought to most likely originate in humans (i.e., carbapenems are not used in food-animals). A study in China found clonally related carbapenem resistant *E. coli* in backyard food-animals, humans, and the environment [76].

Finally, *E. coli* is a diverse species and shows high rates of recombination and HGT. To understand the true role of animal *E. coli* in the AMR crisis, it is necessary to take into account all the biological (population genetics and physiology) aspects of this bacterium and apply WGS, including whole plasmid sequencing. Fortunately, the declining costs of this technology are allowing its implementation in LMICs. The AMR crisis in human medicine is another example where the One Health paradigm is important.

## 4. Conclusions

We suggest that transmission of antimicrobial resistant commensal *E. coli* or AMR genes between *E. coli* from domestic animals and humans occurs frequently, however it is difficult to detect. The diversity of *E. coli* clones and the turn-over rate of *E. coli* clones in the intestines does not facilitate finding relationships between strains in domestic animals, animal products, and humans. The only way to observe this connection in is by sampling humans, animal products, and domestic animals in the same location and during the same period of time. Finding evidence of AMR gene transmission between bacteria in humans and domestic animals is made even more complex as genes frequently move from one plasmid to another. Observing transmission phenomena will likely require that studies collect spatiotemporally matched samples.

## Figures and Tables

**Table 1:** Description of studies that applied next-generation sequencing to study interspecies transfer of *E. coli* or antimicrobial resistance (AMR) genetic determinants.

Study	Advanced Typing Methods ^1^	Spatially Matched Sampling	Temporally Matched Sampling	Focused on Human Pathogens	Strong Evidence of Animal-Human Transmission	Financial Support
De Been, et al., 2014 [39]	+	−	−	+	−	Government
De Been, et al., 2014 [39]	+	+	+	−	+	Government
Hu, et al., 2016 [53]	+	−	−	−	+	Government
Salinas, et al. 2019 [55]	+	+	+	−	+	Government
Ludden, et al., 2019 [70]	+	−	−	+	−	Government
Day, et al., 2016 [71]	+	−	−	+	−	Government, private, NGO
Dorado-Garcia, et al., 2018 [72]	+	−	−	+	−	Government, private
Mainda, et al., 2019 [73]	+	−	+	+	−	Government, private
Falgenhauer, 2019 [74]	+	+	+	−	+	Government
Berg, et al., 2016 [75]	+	+	+	−	+	Government
Li, et al., 2019 [76]	+	+	+	−	+	Government
Loayza, et al., 2019 [77]	+	+	+	−	+	NGO
Liu, et al., 2016 [78]	+	−	−	+	+	Government
Trung et al., 2019 [79]	+	+	+	−	−	Government
Falgenhauer, et al., 2016 [80]	+	−	−	−	+	Government
Reeves, et al., 2011 [81]	+	+	+	+	+	Government
Hedman, et al., 2019 [82]	−	+	+	−	+	Government, NGO
Trung, et al., 2017 [83]	−	+	+	−	+	Government
Valentin, et al., 2014 [84]	−	−	−	−	−	Government

^1^ Advanced method include whole core-genome sequence typing and plasmid sequencing. NGO: non-governmental organization.
